# SARS-CoV-2 Spike Targets USP33-IRF9 Axis *via* Exosomal miR-148a to Activate Human Microglia

**DOI:** 10.3389/fimmu.2021.656700

**Published:** 2021-04-14

**Authors:** Ritu Mishra, Akhil C. Banerjea

**Affiliations:** ^1^ Laboratory of Virology, National Institute of Immunology, Aruna Asaf Ali Marg, New Delhi, India; ^2^ Institute of Advanced Virology, Trivandrum, India

**Keywords:** SARS-CoV-2, COVID-19, neuroinflammation, exosomes, microRNA, deubiquitinase, microglia

## Abstract

SARS-CoV-2, the novel coronavirus infection has consistently shown an association with neurological anomalies in patients, in addition to its usual respiratory distress syndrome. Multi-organ dysfunctions including neurological sequelae during COVID-19 persist even after declining viral load. We propose that SARS-CoV-2 gene product, Spike, is able to modify the host exosomal cargo, which gets transported to distant uninfected tissues and organs and can initiate a catastrophic immune cascade within Central Nervous System (CNS). SARS-CoV-2 Spike transfected cells release a significant amount of exosomes loaded with microRNAs such as miR-148a and miR-590. microRNAs gets internalized by human microglia and suppress target gene expression of USP33 (Ubiquitin Specific peptidase 33) and downstream IRF9 levels. Cellular levels of USP33 regulate the turnover time of IRF9 *via* deubiquitylation. Our results also demonstrate that absorption of modified exosomes effectively regulate the major pro-inflammatory gene expression profile of TNFα, NF-κB and IFN-β. These results uncover a bystander pathway of SARS-CoV-2 mediated CNS damage through hyperactivation of human microglia. Our results also attempt to explain the extra-pulmonary dysfunctions observed in COVID-19 cases when active replication of virus is not supported. Since Spike gene and mRNAs have been extensively picked up for vaccine development; the knowledge of host immune response against spike gene and protein holds a great significance. Our study therefore provides novel and relevant insights regarding the impact of Spike gene on shuttling of host microRNAs *via* exosomes to trigger the neuroinflammation.

## Introduction

SARS-CoV-2 is the latest and seventh strain of the Coronaviruses (CoVs) family, responsible for causing pneumonia like respiratory distress syndrome also termed as COVID-19 in humans (1, 2). SARS-CoV-2 has caused a worldwide pandemic; total number of infections have crossed 116 millions while death numbers have crossed 2.5 millions and still going on (https://covid19.who.int/). SARS-CoV-2 pandemic has posed an unprecedented human health and socio-economic losses (3, 4).

SARS-CoV-2 is a positive-sense, single-stranded RNA virus consisting of a non-structural replicase polyprotein as well as structural proteins such as spike (S), membrane (M), envelope (E), and nucleocapsid (N) proteins (5). SARS-CoV-2 infects both upper and lower respiratory tract epithelial cells and causes from mild flu like symptoms to severe acute respiratory syndrome or septic shocks in severe cases (4). Clinical reports indicate devastating damage to lungs, gut, kidneys, cardiovascular system and CNS in severe cases of SARS-CoV-2 infection (6, 7). Cellular infection by any pathogen triggers host innate and adaptive immunities to contain the pathogen. However in attempt to clear the viral particles from host system, an uncontrolled or inefficient immune response can lead to an array of immunopathology and causes serious systemic inflammatory reaction (8, 9).

Neurological damages by coronaviruses are now an established observation, supported by many experimental and clinical reports (10–12). CNS is potentially susceptible for this virus infection as reports clearly indicate COVID-19 patients suffering from neurological signs such as anosmia (loss of taste and smell), nausea, vomiting, headache and cerebral damages (stroke, encephalitis and degenerative symptoms) (13). Presence of SARS-CoV in brain tissues have also been reported before (14) and it is generally suggested that SARS-CoV-2 enters the brain by both route *via* hematogenous route as well as olfactory bulb through retrograde neuronal travel (10, 15, 16).

ACE2 (Angiotensin Converting Enzyme 2) is the major entry receptor for SARS-CoV-2 (17).

ACE2 is abundantly expressed in the lung-epithelium, small intestine epithelial cells, vascular endothelium, cardiac smooth muscle cells and many other organs including brain (18). Since glial cells and neurons abundantly express ACE2; it raises a strong plausibility that SARS-CoV-2 would target CNS (19). A recent 3D brain organoid study resolved that SARS-CoV-2 targets mainly neurons since ACE2 receptor are present in neurons (20). However they simultaneously also demonstrated that neurons do not support a prolific replication of SARS-CoV-2 (20).

Human brain maintains the homeostasis of the internal system by constantly sensing the signals from periphery and thereby generate a coordinated neural and hormonal responses. Pathogenic invasions either neurotropic or non-neurotropic (virus/bacteria etc.) disrupt this homeostasis by either directly infecting and damaging the CNS or impacting its function in a bystander fashion (21–23). Peripheral infection (viral, bacterial and others) as well as tumors and cancers are known to affect CNS *via* transferring the signals through exosomes and extracellular vesicles (24–27). Many viruses like HIV-1, HCV, HTLV, DENV, ZIKV and others are reported to exploit the exosomal cargo and other extracellular vesicles to execute their pathogenesis (28–32). Among other cargo, microRNAs have gathered special attention in understanding the role of exosomes in modulating host-pathogen interactions. MicroRNAs are 19-21 nucleotide long small regulatory class of RNAs, which suppress their target gene translation *via* binding with their 3’UTR (33). MicroRNAs participate in regulation of almost all cellular and physiological processes of an organism namely developmental transitions, neuronal patterning/functions, apoptosis, cell cycle, metabolism, immune responses, inflammation and host-pathogen interaction etc (34). Viral infections such as Dengue virus infection, ZIKV, JEV, HCV, HIV, herpesvirus, polyomavirus, retroviruses, pestivirus, and hepacivirus are extensively known to play with host microRNA machinery for their successful life cycle (29, 35–37). It have been reviewed in detail elsewhere (33, 34, 38).

Therefore the role of circulating peripheral exosomes, their release and specifically the detail information of exosomal cargo becomes pivotal in understanding the SARS-CoV-2 neuropathogenesis. Exosomes are 30-100 nm sized, small vesicles of endosomal origin and are known to carry myriad of fully functional RNAs, proteins, lipids and microRNAs (39–41). Exosomes and other extracellular vesicles have emerged as a potent mediators during host-pathogen interactions (32, 41). In context of communication between periphery and CNS also, exosomes have become instrumental and play a crucial role during CNS infection and neuroinflammation (25, 30, 42). However literature still lacks any concrete information regarding the role of exosomes during SARS-CoV-2 pathogenesis and neuropathogenesis.

Ubiquitination/deubiquitination is a basic cellular housekeeping process that controls the turnover of all cellular proteins along with regulating multiple protein-protein interactions, protein localization etc (43–45). This makes ubiquitination/deubiquitination process, a preferred pathway to be enormously exploited by many viruses (29, 46, 47). As SARS-CoV-2 is the latest coronavirus, studies exploring the role of ubiquitination/deubiquitination process in overall pathogenesis are still lacking.

The neuropathology of SARS-CoV-2 infection and their mediators for impacting CNS function (since CNS doesn’t support much of SARS-CoV-2 replication) is completely unexplored. Their neurotropism and neuroinvasiveness are the latest burning questions. Since most patients in the current pandemic survive the acute phase of SARS-CoV-2 infection, it is unclear to predict the long-term chronic effects regarding CNS damage. SARS-CoV-2 neuropathogenesis, particularly the roles of their individual proteins are yet to be explored.

In this study, we have examined the function of SARS-CoV-2 Spike protein and its role in manipulating exosomal cargo. The role of exosomes secreted from SARS-CoV-2 spike gene transfected cells, identification of microRNA-148a as a crucial cargo and its regulatory function upon USP33 and downstream targets in human microglia. We have also resolved the novel function of USP33 as a stabilizer of IRF9 protein levels in human microglia and thereby regulating the neuroinflammatory gene expression circuits in CNS.

## Material and Methods

### Cell Culture, Plasmids, and Transfections

HEK-293T and human microglial cell line (CHME3) were cultured in DMEM medium (Himedia #AL219A) with 10%FBS (Himedia #RM1112) and 1% antibiotic/antimycotic solution (Himedia #A002A). Human microglial cells CHME3, have been used for studying inflammatory pathways gene expression and HEK-293T cells have been utilized for luciferase reporter assay, in-vitro-ubiquitination assay, cycloheximide chase assay and for spike gene transfection purposes. All the cells have been grown in 37°C culture-incubator with constant supply of 5% CO_2_. All plasmid DNA transfections have been performed with Lipofectamine 2000 (#11668019, Invitrogen™) as per manual provided with kit.

SARS-CoV-2 Spike gene (S) plasmid (pTwist-EF1α-nCoV-2019-S-2×Strep) was a kind gift from Nevan J. Krogan, QBI COVID-19 Research Group (QCRG), San Francisco, CA 94158, USA. Flag-USP7 plasmid was a kind gift by Altaf Wani (Ohio State University, OH, USA). 6X His-Ubiquitin plasmids were a gift from Prof. Dimitris Xirodimas, University of Dundee, UK. Renilla luciferase construct utilized for normalization process in luciferase reporter assay were given generously by Dr. Vivek Natrajan, IGIB, Delhi, India. NF-kB-Luc and TNFα-luc constructs were obtained from Stratagene. Flag-HA-USP33 (Addgene plasmid # 22601) (48), IFN-beta-pGL3 (Addgene plasmid #102597) (49–51). pLV-IRF9 was a gift from George Stark (Addgene plasmid # 71452) (52–54). psiCHECK IRF9 3’UTR was a gift from Thomas Tuschl (Addgene plasmid # 19863) (48). All the Addgene constructs were commercially purchased from Addgene, USA.

### Exosome Harvesting and Characteristics Analysis

2µg of SARS-CoV-2 Spike gene (S) plasmids (pTwist-EF1α-nCoV-2019-S-2×Strep) were transfected in 1x10^5^ of HEK-293T cells in 6-well plate format with Lipofectamine 2000. Regular passage of transfected cells were done to expand their number and yield of harvested conditioned media from the cells that contained all the secreted extracellular vesicles. Upon every third day, the supernatant were collected from Spike transfected cells. Mock transfected cells were considered as control and supernatants were collected similarly. Supernatants were pooled up and exosomes isolation were performed as per previously established methods described by Miranda et al. (55). In this method, the supernatants were harvested and “salting-out” procedure were performed for the isolation of exosomes. After clearing the cell debris by centrifugation at 2000 rpm, 10% sodium acetate buffer (1 M and pH 4.75) were added to the harvested supernatant and incubated on ice for almost ~60 minutes. A short heating at 37°C for 5 minutes were given which leads to turbid solutions indicating the aggregation of microvesicles. These turbid solutions were then centrifuged at 10000g for 60 minutes to obtain the pellets of extracellular vesicles. These pellets were then washed with 0.1M Na-acetate buffer followed by a final high-speed centrifugation and final exosomes pellets were dissolved in PBS and stored at 4°C till further analysis. Total yield of exosomes were measured as protein content with the help of Bradford reagent. TSG101 protein expressions were considered as tetraspanin marker and have been confirmed in all exosomes preparations. Additionally, CD63 has also been checked as exosomal marker in exosome preparation. Absence of cellular endosomal marker protein ‘Calnexin’ have been confirmed in all the isolated exosomes. These exosomes pellets were then subjected to various miRNA analysis by qPCR and protein analysis *via* western blotting. Human microglial cells CHME3 were treated with various amounts of exosomes (2 µg and 4µg as shown in their respective experiments) for 24 or 48 hours as indicated in individual experiments.

### Cell Lysis and Immunoblot Analysis

Cell lysis have been done with RIPA buffer (20mM Tris [pH 7.5], 150mM NaCl, 1g/mL leupeptin, 1mM-glycerophosphate, 1mM Na3VO4, 2.5mM sodium pyrophosphate,1mM EGTA, 1% NP-40 and 1% sodium deoxycholate). 1X Protease inhibitor cocktail from Sigma (#S8820, Sigma‐Aldrich) was used for inhibiting protein degradation. Bradford assay (#500‐0006; Bio‐Rad Laboratories) has been used for total protein estimation. 30µg of total protein lysates were boiled with 4X loading dye and run on 8-10% polyacrylamide gel at 120 volts. Proteins were transferred on nitrocellulose membrane (#SCNJ8101XXXX101 MdI, advanced microdevices Ltd) by running at 100 Volts for 2 hours in a wet-transfer apparatus. Skimmed milk powder (# GRM1254, Himedia) have been used for blocking the transferred membrane for one hour at room temperature on slow rocker shaker. 5% BSA solution have been used for primary and secondary antibody incubations. Primary antibodies were incubated with membrane for overnight at 4°C. Primary antibody have been given at various dilutions, 1:500 upto1:5000 depending on antibodies and genes in respective experiments described individually in their legends. Membrane received three washings to remove excess primary antibodies followed by one hour incubation with respective secondary antibodies, given in 5% BSA. After 1 hour incubation, excess secondary antibodies were removed with three TBST washings given 15 minutes each. HRP conjugated secondary antibodies for anti-rabbit IgG and anti-Mouse IgG were from Jackson Immunoresearch, USA. ECL western blotting substrate (#32106 Pierce, Thermo Scientific) were used to develop signals on X-ray films (#6568307, Carestream Health Inc., USA). Equal loading of protein samples have been assessed through images density measured by densitometry, performed on ImageJ software version-1.52q. Image densities of GAPDH protein in the respective lanes have been utilized as normalizer in all the western blotting experiments.

### Antibodies and Inhibitors

Antibodies used in this study are; anti-Calnexin, (#2679, Cell Signaling technology), anti-CD63, (#sc-5275), USP42 (sc-390604, Santacruz Biotech), USP33 (#sc-100632, Santacruz Biotech), GAPDH (#sc‐32233, Santacruz Biotech), USP7 (#D17C6, Cell Signaling Technology), TSG101 (#sc-13611, Santacruz Biotech), IRF9 (#76684, Cell Signaling Technology), SARS-CoV-2 Spike Protein S2 Monoclonal Antibody (1A9), # MA5-35946, Invitrogen). Proteasome inhibitor MG132 (#C2211, Sigma‐Aldrich) has been used for *in vivo* ubiquitination assay in HEK-293T cells. Final concentration of 10µM MG132 dissolved in DMSO have been applied on cells for 8 hours. For exosomes biogenesis inhibition, GW4869; a neutral sphingomyelinase (#D1692-5MG, Sigma Aldrich), have been used at 10µM final concentration. For general deubiquitinase inhibition, PR-619 (#SML0430-1MG, Sigma Aldrich) have been used at 10µM final concentration. siRNA for negative control (# 1027280, Qiagen), USP33 (#SI00109123, Qiagen) and IRF9 (# 1027417, Qiagen) were purchased as Flexitube siRNA from Qiagen.

### RNA Extraction and microRNA Assay

RNA extraction was performed with miRNeasy Mini kit (#217004 Qiagen), protocol followed as per manufacture’s instruction. microRNA specific primers have been used for cDNA synthesis provided by TaqMan MicroRNA assay system with TaqMan reverse transcription kit (#4366596, applied Biosystem). Reverse transcription was done at thermal incubations as follows; 16°C for 30 min, 42°C for 30 min, and 85°C for 5 min. MicroRNA qPCR analysis have been done by using universal PCR master mix (#4324018, applied Biosystem). Changes in cellular levels of microRNAs were checked with commercial TaqMan MicroRNA assay (TaqMan Assay ID: 000470 for human miR-148a, # 002677 for miR-590-3p and RNU24 #001001; Applied Biosystem). RNU24 has been used as qPCR internal normalization control. Thermal cycles for qPCR were as follows; 95°C for 10 min, followed by 40 cycles of 95°C for 15 s and 60°C for 60 s. All qPCR reactions have been performed on ABI 7500 fast Real Time thermal cycler from Applied Biosystem.

### Bioinformatics Prediction Tools for microRNA Target Prediction

To search the potential targets of miR-148a and miR-590-3p, microRNA bioinformatics prediction tools such as microRNA.org, miRDB and TargetScan 7.1 have been employed. Complementary binding sites in 3’UTR of IRF9 and USP33 genes for miR-590-3p and miR-148a respectively have been identified with the help of miR-Base and microRNA.org.

### microRNA Mimics and Anti-miRs Transfection

To confirm the IRF9 and USP33 gene regulation *via* microRNAs, miR-148a mimics (# MC10263, Thermo Fisher Scientific), antago-miR-148a (#AM10263, Thermo Fisher Scientific), miR-590-3p mimic (# MC12644, Thermo Scientific) and anti-miR-590-3p (# AM17000) have been used. A day before transfection, human microglial cells, CHME3 were seeded at 1x10^5^ cells in each well (6-well plate format). 100 picomoles of mimics and anti-miR-148a were transfected per well with Lipofectamine RNAi Max transfection reagent (# 13778150, Invitrogen) as per manufacturer’s instructions. CHME3 cells were harvested post 48 hours of transfection and proceeded for RNA and western blot analysis.

### Dual Luciferase Reporter Assay

HEK-293T cells have been utilized for all the luciferase assays. Promoter activity assays for TNFα, IFN-β and NF-κB were done by co-transfection methods in 6 well plate format. One day before transfection, 1x 10^5^ cells were seeded to reach almost 60% confluency at the time of transfection. 1µg TNFα, IFN-β and NF-κB plasmids were co-transfected with 1µg of Spike plasmids, USP33 plasmids and IRF9 plasmids. Similarly in IRF9 siRNA experiments, 100 picomoles of siRNA against IRF9 were transfected 12 hours before TNFα, IFN-β and NF-κB plasmids transfections. For transfection normalization, 500 ng of Renilla luciferase plasmids were transfected in all wells. Post 24 hours of transfection, HEK-293T cells were harvested and proceeded for dual luciferase assay as per manufacturer’s instructions (# E1910, Promega). Synergy H1 multi-mode reader, Biotek have been employed for capturing luminescence readings followed by capturing Renilla luminescence in all the wells. Renilla luminescence reading have been used as denominator to get the final luciferase promoter activities.

### *In Vivo* Ubiquitination Assay

In vivo ubiquitination assay was performed as described in our previous publication (29). Briefly, HEK-293T cells were seeded in 90 mm culture dishes to reach for almost 70% confluency before transfection. 5µg of pLV-IRF9 plasmids and 5µg of 6X-His-ubiquitin plasmids were transfected along with 5µg of Flag-HA-USP33 in co-transfection experiments to check the impact of USP33 on ubiquitination levels of IRF9. After 24 hrs of transfection, all cells were treated with 20µM final concentration of MG132 and incubated for at least 8 hours. Cells were then harvested and sonicated and lysed with Buffer A (6M guanidine-HCL, 0.1M Na2HPO4, 10mM imidazole at pH 8.0). The lysates were then incubated with Ni-NTA agarose beads overnight on rotor shaker at room temperature. The beads were washed next morning with buffer A, followed by Buffer A+Ti (1 volume of buffer A and 3 volume of buffer Ti (25mM Tris-HCL, 20mM imidazole at pH 6.8) and finally with buffer Ti. Final elutions were done with 50µl of His-Ubiquitin elution buffer (0.2M imidazole, 5% w/v SDS, 0.15M Tris-Cl at pH 6.8) also known as 2X Laemmli buffer and boiled for 5 minute at 100°C. Samples were run on SDS gel and probed with anti-IRF9 antibody.

### Half-Life/Cycloheximide Chase Assay

Cycloheximide is a well-known translation inhibitor to analyze the half-life of cellular proteins. HEK-293T cells were used for cycloheximide chase assay in 6 well plate format with 1x10^5^ cells per well. At ~70% confluency, cells were transfected with IRF9 only and IRF9 plus USP33 co-transfection in different plates with the help of Lipofectamine 2000. Post-24 hrs of transfection, Cycloheximide (#01810, Sigma-Aldrich) were given to all wells at 100µg/mL concentration. Cells were then harvested at various time points (2, 4, 8, 12 and 24 hours) followed by lysis with RIPA buffer and western blot analysis for indicated proteins.

### Statistical Analysis

All the experiments have been repeated independently as three biological replicates to obtain means, standard deviation and standard error of means (S.E.M). The levels of significance (p values) have been obtained *via* student’s t-test and one-way ANOVA whichever applicable. p<0.05 have been taken as statistically significant and displayed as * or ** in their respective experiments. Real time (qPCR) results are displayed as relative changes in miR quantity (RQ), calculated by double delta Ct (ΔΔCt) algorithms.

## Results

### Spike Transfected Cells Release Exosomes Loaded With miR-148a and miR-590

**Figure 1:**
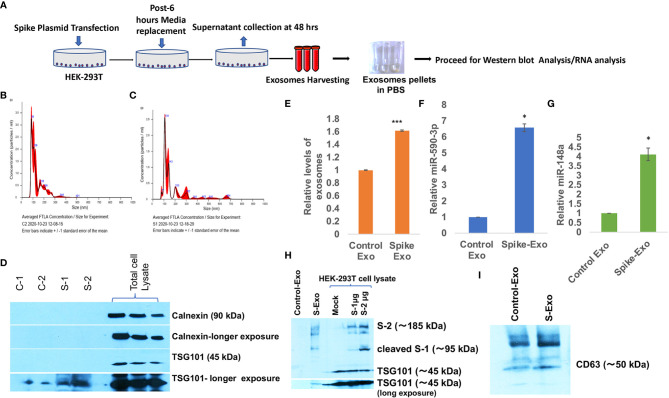
Spike transfected cells released exosomes are loaded with miR-148a and miR-590. **(A)** Work-flow schematic depicting exosome harvesting protocol from Spike gene transfected HEK-293T cells. **(B)** Size distribution analysis graph of control exosomes done on NanoSight, NTA 3.2 Dev Build 3.2.16, indicating mode of size range 90.6 nm. **(C)** Size distribution analysis graph of S-exosomes, indicating mode of size range of 107.5 nm. **(D)** Immunoblot image for characterization of harvested exosome preparations. Endosomal protein Calnexin is absent in exosome population indicating the purity of exosome and free of cellular debris. TSG101 as tetraspanin marker protein are present in exosomes. **(E)** Graph bars showing relatively higher amount of exosome secretion by Spike transfected cells, measured as total protein *via* Bradford assay. **(F)** Graph bars displaying relative miR-590 levels in Spike-Exosomes compared to control exosomes. **(G)** Graph bars displaying relative levels of miR-148a in Spike-Exosomes as compared to control exosomes. Exosomes pellets were subjected to total RNA isolation and microRNA levels measured through qPCR analysis *via* TaqMan assay specific for miR-590 and miR-148a respectively. **(H)** Western blot image, confirming the Spike gene transfection in HEK-293T cells as well their accumulation in secreted exosomes. 1µg and 2 µg of Spike gene (S) plasmids (pTwist-EF1α-nCoV-2019-S-2×Strep) were transfected in 6-well plate format for 24 hours with the help of Lipofectamine 2000. **(I)** Western blot image showing CD63 in harvested exosomes as exosomal marker. All the experiments have been repeated at least three times independently to obtain mean and mean ± S.E.M. Levels of significance have been calculated by student’s t-test and statistical significance indicated as single * for p values <0.05 and *** for p values <0.0005.

Coronavirus spike protein (S Protein) is the outermost ‘crown like’ structural protein that mediates coronavirus entry into the host cell (56). Coronavirus spike gene have been the prime target for developing an effective vaccine and therapy. Since S protein is supposed to play a crucial role in inducing neutralizing antibody, T-cell response and protective immunity (57–59); we were interested to evaluate the impact of spike protein in perturbing the immune response specifically in context of CNS. We transfected HEK-293T cells with S gene plasmids (pTwist-EF1α-nCoV-2019-S-2×Strep) and harvested the released exosomes on every 24 hours, pooled them and followed the protocol of exosome isolation as displayed in schematic (**Figure 1A**). Exosome characterization were performed as per established standards. The particle size distribution analysis showed that harvested exosomes from control and S gene transfected cells were in size range of a typical exosomal population (mode: 85.9 nm for control exosomes, mode: 107.2 nm for S exosomes (**Figures 1B, C** **)**. Purity of exosomes and exclusion of cellular debris were checked by absence of endosomal protein Calnexin in exosome population (**Figure 1D**) and tetraspanin marker protein TSG101 were also confirmed in harvested exosomes (**Figure 1D**). We have also confirmed the presence of another exosomal marker CD63 in harvested exosomes (**Figure 1I**). Confirmation of SARS-CoV-2 spike protein expression in transfected HEK-293T cells were also done (**Figure 1H**). Disruption of exosomal cargo and manipulation of target recipient cell signaling by SARS-CoV-2 is a completely unexplored area. This prompted us to examine the potentially loaded cargo in the form of microRNAs, mRNA or proteins. We chose to focus on actively loaded microRNAs in these released exosomes because viral infection and inflammation nexus are intricately related with modulation of microRNAs (34). Viral refashioning of host microRNAs that target the expression levels of inflammatory gene expressions are a well-known phenomenon (33, 34, 37, 38, 60). Levels of total secreted exosomes were significantly higher from S-transfected cells compared to mock transfected cells (**Figure 1E**). We observed a significant higher loading of miR-590 (~ 6.6 folds) and ~4.2 folds higher loading of miR-148a in S-transfected cell released exosomes (**Figures 1F, G** **)**.

### Spike Transfected Cells Released Exosomes Suppress USP33 and IRF9 in Recipient Human Microglia

**Figure 2:**
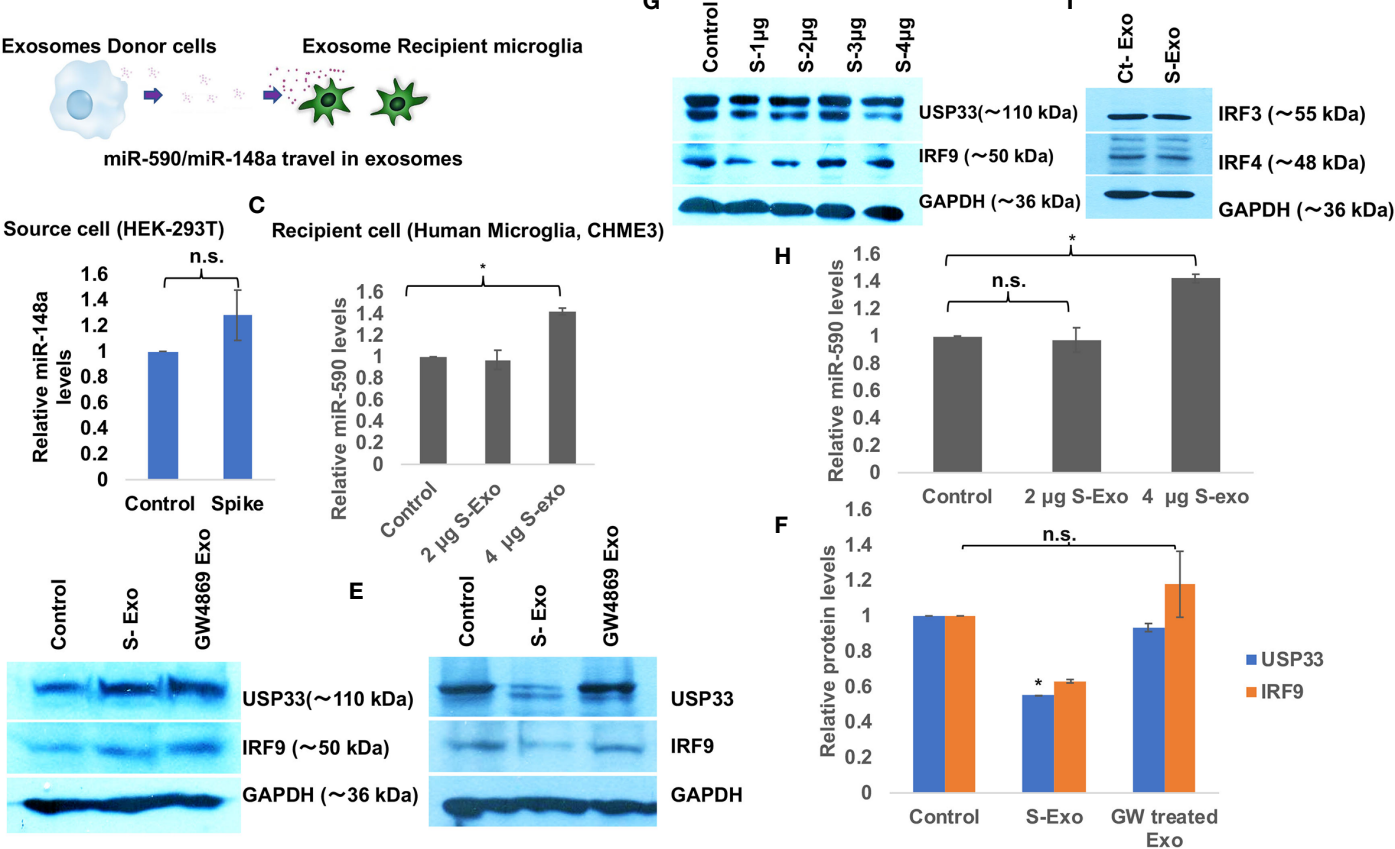
Spike transfected cells release exosomes to suppress USP33 and IRF9 in human microglia **(A)** Schematic to demonstrate the Spike transfected HEK-293T cells as exosome donor cells and human microglia CHME3 are recipient cells for those released exosomes. These exosomes carry miR-148a and miR-590 as their cargo. **(B)** Graph bars showing levels of miR-148a in source cell i.e. HEK-293T cells after Spike transfection. **(C)** Graph bars indicating the increase in miR-148a levels in a dose dependent fashion in recipient human microglia. **(D)** Immunoblot images showing the cellular levels of USP33 and IRF9 in exosome source cells (spike transfected HEK-293T cell). **(E)** Immunoblot images showing decrease in USP33 and IRF9 expression levels in human microglia after receiving S-exosomes and GW-4869 treated exosomes from Spike transfected HEK-293T cells. **(F)** Graph bars showing average change in USP33/IRF9 levels after S-exo and GW-4869 treated exosomes. Densitometry analysis of western blot images were done on ImageJ 1.52q version software. GAPDH lane density have been used as normalizer for all image densitometry analysis. **(G)** Western blot image showing changes in USP33 and IRF9 levels after direct transfection of spike gene plasmids at different doses in microglia cell. **(H)** Graph bars showing relative miR-590 levels in recipient microglia cells after treatment with two doses of exosomes. **(I)** Western blot image showing almost no change in IRF3 and IRF4 gene expression levels upon S-exo treatment on human microglial cells. All the experiments have been biologically repeated at least three times to get the average change in expression levels. All graph bars are showing mean and mean ± S.E.M. student’s t-test have been done to get the statistical significance indicated as single * for p values <0.05.

We wanted to understand the bystander impact of SARS-CoV-2 infection on disruption of CNS homoeostasis. Multiple organ dysfunctions including neurological sequelae during SARS-CoV-2 infection is a widespread observation even when the organ is not directly infected with the virus. To dissect this scenario further, we have used exosome released from spike transfected cells instead of using total SARS-CoV-2 infection. We transfected Spike gene in HEK-293T cells and harvested the released exosomes, which we found full of miR-148a and miR-590 among other cargo (**Figures 1E–G**). We treated human microglia with these loaded exosomes for 24 hours and assessed the protein expression levels of target genes USP33 and IRF9 (**Figure 2A**). USP33 and IRF9 were chosen because they emerged as the potential target of miR-148a and miR-590 respectively, in multiple bioinformatics prediction tools. We checked the miR-148a and miR-590 levels in both, the donor cells (S-transfected HEK-293T cells) and recipient human microglia. In donor cells, level of miR-148a were found slightly higher by only 1.3 folds (**Figure 2B**) indicating that miR-148a is actively loaded in released exosomes. At two different doses of 2µg and 4 µg of S-Exo treatment, level of miR-148a were significantly increased up to 3.5 folds and 16 folds respectively (**Figure 2C**) in recipient human microglia. However, levels of miR-590 in recipient microglia increased only slightly at 4 µg of S-Exo treatment (**Figure 2H**). The cellular levels of USP33 and IRF9 in S-transfected cells (exosomes donor) were found slightly increased compared to control cells (**Figure 2D**).

The cellular expression levels of both targets USP33 and IRF9 were decreased up to 50% and 60% in S-Exo treated human microglia (**Figures 2E, F** **)**. S-transfected HEK293T cells were also treated with GW4869, a pharmaceutical inhibitor of neutral sphingomyelinase, which blocks the inward budding of microvesicles and thereby generation of exosomes. When such blocked conditioned media were used as treatment on recipient microglia, they couldn’t decrease the levels of USP33 and IRF9 (**Figures 2E, F** **)**. This confirmed our idea that released S-Exo is largely responsible for modulating USP33 and IRF9 expression levels. When human microglia were directly transfected with S gene plasmids, the expression levels of USP33 and IRF9 remained largely unaffected (**Figure 2G**).

### miR-590 Directly Targets IRF9 Expression Levels

**Figure 3:**
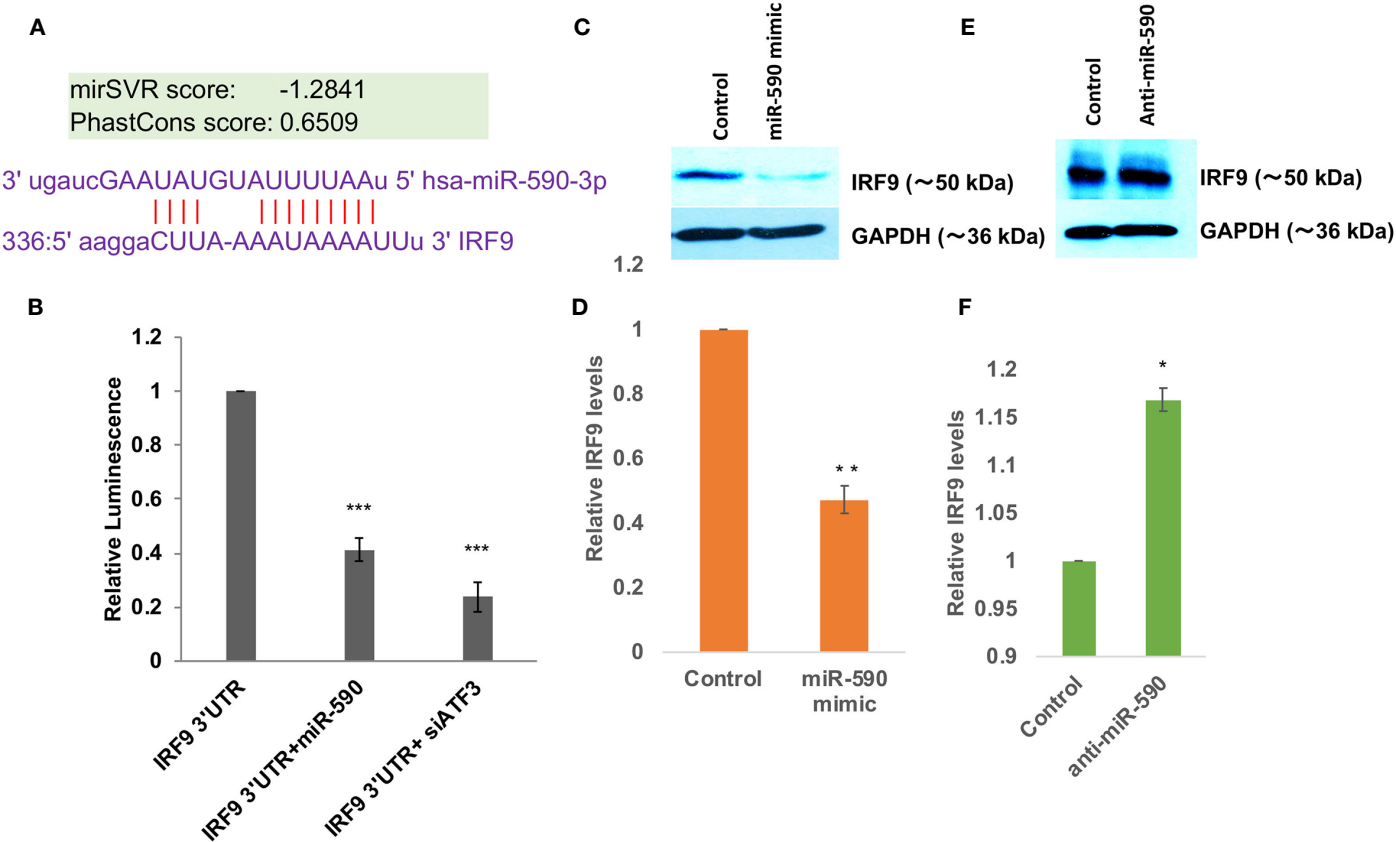
miR-590 directly targets IRF9 expression levels **(A)** Image showing complementary binding between 3’UTR of IRF9 and seed sequences of miR-590. **(B)** Graph bars showing results of dual luciferase assay for establishing direct binding and targeting of 3’UTR of IRF9 gene by miR-590. Dual luciferase assay were performed by co-transfection methods in HEK-293T cells in 6 well plate format, lysed and measured with Promega luciferase assay kit. Renilla luciferase plasmids have been co-transfected for normalization and getting true changes in luciferase values. **(C)** Immunoblot image showing decrease in IRF9 expression levels after miR-590 mimic transfection in CHME3 cells. Lipofectamine RNAiMax have been used for miR-590 mimic transfection in 6 well plate format with 1x10^5^ cells at the time of transfection. **(D)** Graph bars displaying densitometry analysis for average IRF9 level change after miR-590 overexpression. **(E)** Immunoblot image showing rescued expression levels of IRF9 upon anti-miR-590 transfection in human microglial cell line CHME3. 100 picomoles anti-miR-590 were transfected with Lipofectamine RNAiMax reagent in 6 well plate format with 1x10 ^5^ cell. **(F)** Graph bars showing average change in IRF9 levels after anti-miR-590 transfections. All the experiments have been independently repeated three times to get the average changes in expression levels. Graph bars are displaying mean and mean ± S.E.M. student’s t-test have been performed to obtain the statistical significance indicated as single * for p values <0.05, ** for p values <0.005 and *** for p values <0.0005.

The direct targeting of USP33 protein expression by exosomal miR-148a were established in our earlier study (29). So we focused for investigating miR-590 mediated regulation of IRF9. Bioinformatics prediction tools such as TargetScan, MicroRNA.org, miRDB; all suggested a potential binding site of miR-590 seed sequences onto 3’UTR of IRF9 (**Figure 3A**). The mirSVR score were found as -1.2841 suggesting a strong free energy change upon this binding which means a strong binding affinity between miR-590 and complementary IRF9 3’UTR sequences. We performed a luciferase reporter assay for this target validation. Co-transfection of IRF9 3’UTR and miR-590 mimics showed a significantly reduced luminescence (~60%) suggesting a binding and blockage of luciferase expression (**Figure 3B**). On the other hand, ATF3, a transcription factor and negative regulator of miR-590 (61), were also blocked with siRNA against ATF3, to elevate the cellular levels of miR-590. In this co-transfection, elevated miR-590 again suppressed the luminescence activity, which suggested that IRF9 expression level is indeed regulated by miR-590 (**Figure 3B**). miR-590 overexpression with the help of commercial miR-590 mimics significantly reduced the target IRF9 protein expression levels (~50%) (**Figures 3C, D** **)**. Contrary to that, transfection of anti-miR-590 significantly rescued the expression levels of IRF9 (**Figures 3E, F** **)**. These standard validation experiments confirmed the regulatory relationship between miR-590 and IRF9 gene.

### USP33 Regulates IRF9 Turn-Over in Human Microglia

**Figure 4:**
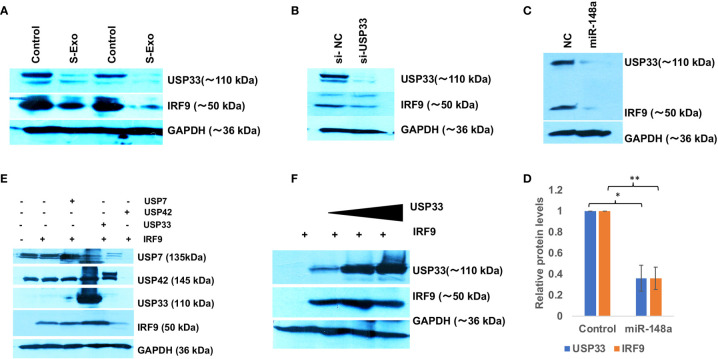
USP33 stabilizes IRF9 levels in human microglia. **(A)** Western blot analysis of USP33 and IRF9 expression levels in CHME3 cells upon treatment with 2µg of S-exosomes. CHME3 cells were seeded at 1x10^5^ density in 6 well plate format, were serum starved for 1 hour before exosomes treatment. Cells were harvested post-48 hours treatment of exosomes, lysed with RIPA buffer and processed for western blot analysis. **(B)** Immunoblot image showing the impact of depletion of USP33 on cellular levels of IRF9. siRNA against USP33 were transfected in CHME3 cells, at 100 picomoles per well in 6 well plate format with Lipofectamine RNAiMax reagent. After 48 hours cells were harvested, lysed with RIPA buffer and processed for western blotting. **(C)** Immunoblot image showing reduced levels of USP33 upon miR-148a mimic transfection and its direct influence on IRF9 levels in CHME3 cells. miR-148a mimic were transfected at 100 picomole concentration in CHME3 cells at 1x10^5^ cells density in 6 well plate format. **(D)** Graph bars showing average change in USP33 and IRF9 levels after miR-148a mimic transfection. Densitometry analysis of western images were performed on Image J software version 1.52q. Data are displayed as mean and mean ± S.E.M. from three independent experiments. Student’s t-test have been applied to get the statistical significance, indicated as ** for p values <0.005. **(E)** Immunoblot analysis showing the specific deubiquitinase activity of USP33 over IRF9 stabilization. HEK-293T cells have been used for co-transfection experiments in 6 well plate format at 1x10^5^ density. 1µg of IRF9 plasmids have been co-transfected with 1µg of USP33, 1µg of USP42 and 1µg of USP7 in various wells. After 24 hours, cells were harvested and lysed in RIPA and followed by western blot analysis. **(F)** Immunoblot image showing dose dependent impact of USP33 mediated stabilization over IRF9 levels. HEK-293T cells have been co-transfected with 1µg IRF9 plasmids with indicated amount of USP33 plasmids (1µg, 2µg and 3µg). After 24 hours, cells were harvested and proceed for western blotting analysis. All the experiments have been independently repeated at least three times to reach the conclusion.

We initially hypothesized that miR-590 loaded in S-exo would be internalized and significantly elevate the levels of miR-590 in recipient human microglia and therefore would reduce the target IRF9 expression levels. However, qPCR analysis of miR-590 in S-exo recipient microglia showed that upon 2 µg of S-Exo treatment, there was not enough elevation in miR-590 levels in recipient microglia (**Figure 2H**). A small increase in miR-590 in recipient microglia was only observed at 4 µg of S-Exo treatment (**Figure 2H**). This forced us to consider additional regulatory pathways which might be operating prominently in downregulating IRF9 levels in S-exo treated human microglia. Simultaneously, we observed a consistent trend that cellular levels of IRF9 was always following the cellular levels of USP33 in human microglia. Upon S-exo treatment, USP33 levels went down, followed by similar downregulation in IRF9 protein levels (**Figure 4A**). We blocked the cellular levels of USP33 by transfecting siRNA against USP33 and we were able to observe a reduced levels of cellular IRF9 levels (**Figure 4B**). We also transfected human microglia with exogenous miR-148a mimic, which we earlier observed to be loaded and transported within S-exo. Downregulation of USP33 *via* miR-148a mimic were also followed by reduced levels of IRF9 in human microglia (**Figures 4C, D** **)**. This gave us a primary clue that IRF9 levels and its stability might be under the regulation of USP33, which is a deubiquitinase protein. To confirm this regulatory role of USP33 over IRF9, we performed further experiments. We overexpressed USP33 with Flag-HA-USP33 plasmid. Along with, we also transfected some other USPs, such as USP42, USP7. We observed a specific impact of only USP33 on IRF9 stabilization (**Figure 4E**). USP42 and USP7 were not able to stabilize or increase the cellular levels of IRF9 protein (**Figure 4E**). We also gave different increasing doses of USP33 (1µg, 2µg and 4µg) and observed a dose dependent stabilization of IRF9 protein levels in co-transfection experiments (**Figure 4F**). This confirmed the positive regulatory function of USP33 upon IRF9 protein levels.

### USP33 Controls the IRF9 Turnover *via* Its Deubiquitylation

**Figure 5:**
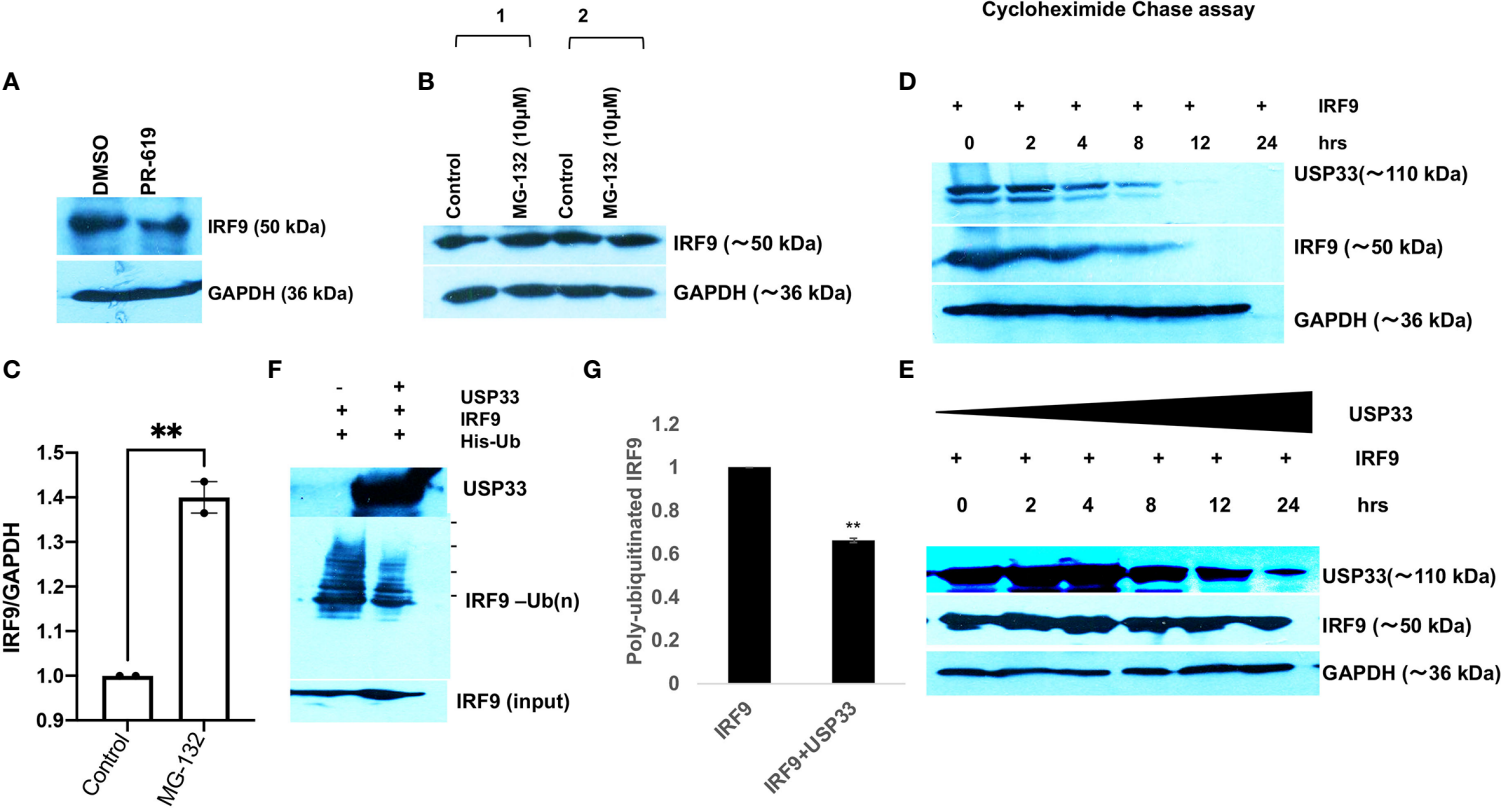
USP33 regulates the IRF9 turnover *via* its deubiquitylation in microglia. **(A)** Immunoblot image showing the impact of deubiquitinase inhibitor PR-619 on the levels of IRF9 in microglial cells. PR-619 were treated at 10 µM final concentration for 8 hours and cells were harvested and followed by western blot analysis. **(B)** Immunoblot images showing the impact of proteasome inhibitor MG-132 on the elevated stability of IRF9 in microglial cells. **(C)** Graph bar displaying the average increase in IRF9 levels upon MG-132 treated human microglial cells. **(D)** Immunoblot analysis of half-life of IRF9 protein measured *via* cycloheximide chase assay done in HEK-293T cells. **(E)** Immunoblot analysis of impact of USP33 on turnover of IRF9; measured *via* cycloheximide chase assay performed in HEK-293T cells. Cycloheximide chase assay have been repeated two times and representative results are displayed here. **(F)** Immunoblot image showing the impact of USP33 on polyubiquitinated levels of IRF9. 5µg of pLV-IRF9 plasmids and 5µg of 6X-His-ubiquitin plasmids were transfected along with 5µg of Flag-HA-USP33 in co-transfection experiments (in 60mm dish format) to check the impact of USP33 on ubiquitination levels of IRF9. After 24 hours, cells were treated with MG132 for 8 hours, followed by *in vivo* ubiquitination assay as described in detail in *Material and Methods* section. **(G)** The graph bars showing the quantified levels of poly-ubiquitinated IRF9. The ubiquitination assay was performed three times independently and average values are shown as graph bars. Data are displayed as mean and mean ± S.E.M. student’s t-test have been utilized to obtain the statistical significance, indicated as ** for p values <0.005.

USP33, Ubiquitin specific peptidase 33 is a deubiquitinase enzyme (DUBs). By virtue of its deubiquitinase activity, it can remove the ubiquitin tagging and thereby stabilize its specific target proteins. We applied a cell-permeable pyridinamine class broad-spectrum DUB inhibitor, PR-619 on microglial cells and tested the cellular levels of IRF9. IRF9 levels were significantly decreased upon PR-619 treatment at 10µM final concentration (**Figure 5A**). We also treated microglial cells with MG132 (a proteasome inhibitor) at 10µM concentration for 8 hours and found the increased or stabilized cellular levels of IRF9 in microglial cells (**Figure 5A**) This strengthens our hypothesis that cellular IRF9 levels are under control of proteasomal degradation machinery and deubiquitinase enzymes play a vital role in maintaining the IRF9 stability. To establish the function of USP33 as IRF9 stabilizer, we performed multiple experiments. A chase assay with cycloheximide were performed to assess the half-life of IRF9. A co-transfection of USP33 and IRF9 were done along with solo IRF9 overexpression in HEK-293T cells in 6-well plate format. IRF9 solo transfection experiment showed that IRF9 half-life ranges between 3-4 hours (**Figure 5D**). USP33 co-transfection significantly improved the turnover time of IRF9 protein inside cells (**Figure 5E**). IRF9 levels were stabilized for up to 24 hours and beyond (**Figure 5E**).

Since our data suggested a positive regulation of cellular IRF9 levels by USP33, we tested the impact of USP33 on ubiquitination levels of IRF9 protein *via in vitro* ubiquitination assay. We found a significant decrease in ubiquitinated IRF9 levels when co-transfected with USP33 (**Figures 5B, C** **)**. These results confirmed the role of deubiquitinase USP33 in stabilizing the IRF9 protein levels. These results explain our previous observations of why IRF9 protein levels were concomitantly changing with changing cellular levels of USP33. Any cellular change of USP33, be it by siRNA-USP33, miR-148a overexpression, plasmid mediated overexpression of USP33 and Spike-exosomes mediated downregulation of USP33, they all showed a downstream alterations in IRF9 protein level.

### SARS-CoV-2 Spike Activates Cytokine Expressions

**Figure 6:**
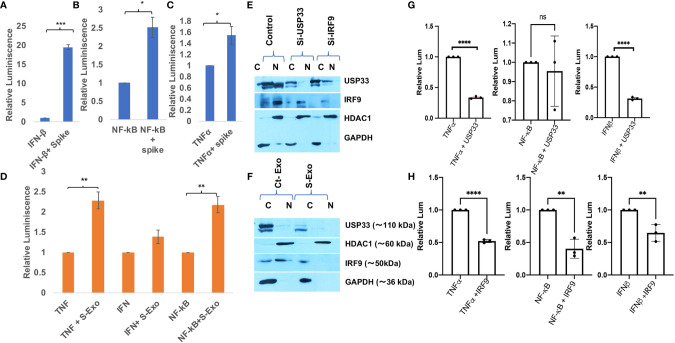
Impact of Spike protein on host cytokine gene expressions. **(A–C)** Impact of direct Spike gene transfection on promoter activity of IFN-β, NF-κB and TNFα. These three plasmids (1µg each) were co-transfected with 2µg of Spike gene plasmids in HEK-293T cells and after 24 hours, cells were harvested and dual luciferase assay were performed. 500 ng of Renilla luciferase plasmids were also transfected and their luciferase values have been used as normalizer in all the experiments. **(D)** Graph bars showing the result of treatment of S-exosomes on the promoter activity of TNFα, IFN-β and NF-κB. Luciferase assay were done in HEK-293T cells as described in previous experiment. **(E)** Immunoblot analysis showing lesser concentration of IRF9 in nuclear fraction of S-exo treated microglia cells. **(F)** Immunoblot images showing the impact of USP33 siRNA and IRF9 siRNA on nuclear concentration of IRF9. **(G)** Graph bars showing relative luminescence in USP33 overexpressed cells. 1µg of USP33 plasmids were co-transfected with 1µg of all three plasmids (IFN-β, NF-κB and TNFα) in their respective wells. **(H)** Graph bars showing relative luminescence in IRF9 overexpressed cells. 1µg of IRF9 plasmids were co-transfected with 1µg of all three plasmids (IFNβ, NF-κB and TNFα) in their respective wells. After 24 hours, cells were harvested and dual luciferase assay were performed. Dual luciferase assays were performed at least three times to get the average values and are displayed as graph bars. Results shown as mean and mean ± S.E.M. Student’s t-test have been used to calculate the statistical significance, indicated as * means p values <0.05, ** for p values <0.005, *** for p values <0.0005, **** for p values <0.00005.

IRF9 expression levels have been considered as an important determinant of viral disease severity (49–51). Previous studies have demonstrated the crucial role of IRF9 in inflammation (62), autoimmune diseases like SLE (63), cardiovascular diseases (64), cell proliferation and immune cell regulation (65). In our experiments, we observed a sharp decline in IRF9 expression levels upon S-exo treatment in human microglia. Considering its multifunctional roles especially for inflammation and autoimmune regulation, we were interested to resolve its specific role in controlling inflammatory gene expression pathways such as NF-κB, TNFα and IFNβ. Firstly, we performed Spike gene transfection along with three major promoter luciferase plasmids of TNFα, IFNβ and NF-κB in a co-transfection experiment and checked the reporter luciferase expression levels as an indicator of these promoter activities. SARS-CoV-2 Spike gene transfection were able to induce all the three cytokine regulatory promoter activities of NF-κB, TNFα and IFNβ (**Figures 6A–C**). We mimicked our experimental set up of treatment with S-exo on cells and checked the promoter activities of these cytokine pathways. We could observe a significant elevation in promoter activity of all three pathways namely NF-κB, TNFα and IFNβ (**Figure 6D**). Since S-exo treatment on human microglia were causing the reduction in cellular IRF9 levels; we checked its nuclear translocation and we could observe a significant reduction of IRF9 nuclear concentration too (**Figure 6E**). In S-exo treated CHME3 cells, IRF9 levels were reduced in nuclear fraction (**Figures 6E, F** **)**. Upon siRNA-USP33 and siRNA-IRF9 transfection too, its concentration in nucleus were found affected (**Figures 6E, F** **)** which would affect the transcriptional activation of its downstream cytokine expression levels. USP33 and IRF9 axis is supposed to control the inflammation as well as anti-viral state of cells. For investigating the impact of USP33 and IRF9 upon inflammatory gene regulatory network, we overexpressed USP33 and IRF9 in separate experimental set-up. Overexpression of USP33 and IRF9 both were significantly suppressing the promoter activities of NF-κB, TNFα and IFNβ (**Figures 6G, H** **)**.

## Discussion

Majority of clinical reports from COVID-19 patients suggest that patients deterioration happens 7-10 days after the onset of disease, which is accompanied by decrease in viral load (66). This suggests that pathological manifestations of COVID-19 are primarily driven by hyperinflammation leading to multi-organ dysfunctions rather than direct viral injury. Previous episodes of coronavirus infections such as SARS-CoV and MERS-CoV have been shown to cause an uncontrolled, tissue-damaging inflammatory phenomenon, also known as *‘Cytokine storm’* (67, 68). The ‘Cytokine storm’ phenomenon have been linked with severity of many viral diseases such as Influenza (IAV) (69), Dengue hemorrhagic fever (70) and Ebola viruses (71) etc.

These information laid the foundation for our hypothesis, where we wanted to investigate the triggering factor for cytokine storm even when whole virus count is declined or even disappeared from host circulation. We specially chose to investigate the immune-modulatory functions of the Spike protein of SARS-CoV-2 since it is the outermost structural protein that interacts with host cell while infecting it. Another reason was its prominent use as effective epitope for vaccine development, which demands the more detail dissection of host inflammatory responses against Spike gene. Apart from acting as instrument for virus entry, Spike has been regarded as critical determinant of host immune responses, tissue tropism and influencing host range for viral transmission (56).

Recent report by Ramani et al. (20), were suggesting that SARS-CoV-2 replication might not be equally potentially supported in all tissues as in lungs despite the presence of ACE2 receptors (20). In this study, authors showed in a 3D brain organoid model that SARS-CoV-2 were infecting neurons but were not replicating efficiently, yet there were enough neuronal damage similar to neurodegenerative phenotype (20). Even some clinical reports are indicating multiple signs of neurological damages in otherwise asymptomatic COVID-19 patients (72, 73). These reports strongly indicate that not just SARS-CoV-2 viral particle but shed viral proteins or ‘toxic trails’ after SARS-CoV-2 can induce a cascade of strong host immune response. These leftover ‘toxic trail’ after viral reclining phase often includes cellular transcription factors, microRNAs and other circulating factors in host plasma. These reports influenced our experimental design and we were curious to look into the role of exosomes for transmitting and transferring the cellular and viral signals during the course of SARS-CoV-2 neuropathogenesis.

Our results (**Figures 2** and **3**) are clearly indicating that Spike transfected cells release a significant amount of exosomes (S-exo), actively loaded with inflammation promoting microRNAs such as miR-590, miR-148a etc. We have chosen human microglia to study the impact of SARS-CoV-2 spike protein induced disruption of CNS innate immune responses. Human microglia, the ‘brain-resident macrophages’ are rightly considered the real executor of neuroinflammation since their role in causing neuroinflammation in various viral diseases (HIV-1, JEV, Dengue etc) are well established (29, 37, 74, 75). Upon exposure with S-exo cargo, human microglia internalizes its cargo such as microRNAs, which ultimately results in suppression of its target genes. In our results, we could show that cellular USP33 levels (a potential target of miR-148a) gets significantly diminished (**Figure 2E**). We have previously established a regulatory axis run by miR-148a mediated targeting of USP33 and downstream regulation of ATF3 turnover during DENV neuropathogenesis (29). Role of miR-148a in congenital ZIKV infection, targeting TLR3 during Duck tembusu virus (DTMUV) and playing their role in tumor invasion and migration are well known (76–78). Similarly diverse roles of USP33 in deubiquitinating Parkin gene, HERC2, centrosome biogenesis, tumor progression of gastric carcinoma as well as DENV neuropathogenesis are well established (29, 79–81).

Since our S-exo cargo also carried a huge amount of miR-590, we checked for the target of miR-590. Bioinformatics prediction tools suggested IRF9 to be a potential target of miR-590. As expected, S-exo treatment on human microglia significantly reduced the cellular expression levels of IRF9 (**Figure 2**). Application of exosome release inhibitor GW-4869 on exosome donor cells (Spike transfected HEK-293T cells), followed by harvesting of exosomes from that source and exposing them on microglia confirmed our results that exosome are the primary source for decline in USP33 and IRF9 levels in human microglia upon S-exo treatment. We could also validate the regulatory interaction of miR-590 over IRF9 by standard microRNA mimic, anti-miR transfections and luciferase reporter assay (**Figure 3**). We also observed the interesting trend in exosome recipient human microglia; the levels of miR-148a were highly increased as compared to miR-590 levels (**Figures 2C, H** **)**. However the target gene expression levels of USP33 and IRF9 were decreased in almost same range (**Figures 2E, F** **)**. IRF9 seemed to follow the cellular levels of USP33 (**Figure 4**). We examined this trend by multiple experiments; where we just manipulated the cellular level of USP33 (by siRNA, miR-148a mimic, USP33 plasmid overexpression etc). We observed that cellular IRF9 levels were always concomitant with the cellular USP33 levels. This indicated a role of USP33 in turnover or stability of IRF9. Since USP33 is a deubiquitinase, its primary function is to remove ubiquitin from its target proteins and stabilize them. We also confirmed the specificity of USP33 over IRF9 stabilization, since no other irrelevant USP42, USP7 were able to stabilize the cellular levels of IRF9 (**Figure 4B**). By using cycloheximide chase assay and *in vitro* ubiquitination assay, we could successfully establish that turnover time or half-life of IRF9 is under the influence of USP33 (**Figure 5**).

IRF9 is relatively less explored member of IRF family, therefore once dubbed as “The forgotten IRF” by Paun and Pitha (82). Available literature suggest that IRF9 is the only IRF that is not regulated by phosphorylation. Most of other IRF’s function are known to be prominently regulated *via* their phosphorylation (30–32, 83). IRF9 combines with phosphorylated STAT1 and STAT2 to form a heterotrimeric transcription complex also termed as ISGF3 interferon-stimulated gene factor 3. This ISGF3 complex moves to nucleus to bind with ISRE (interferon-stimulated response element) (ISRE) and modulates the transcriptional activity of interferon-regulated genes (IRGs) (84).

In literature, it has been emphasized that viruses are evolved to interfere and tackle with IRF9 in multiple ways (61, 85–87). Viruses can antagonize IRF9 *via* blocking its nuclear sequestration, blocking its binding with DNA and *via* promoting its degradation. For example, Human papillomavirus 16 gene, also known as E7 oncogene directly interact with IRF9 and thereby prevent its usual complex formation with STAT1/STAT2 and its nuclear translocation (88). Another study shows that varicella-zoster virus ORF63 protein degrades IRF9 *via* proteasomal degradation pathway (89). In yet another way, Porcine bocavirus NP1 protein were shown to directly bind with DNA binding domain of IRF9 and therefore effectively blocking the ISGF3 complex to bind with target DNA and attenuating the induction of downstream transcriptional activities (90). However, the mysterious duality of IRF9 function in generating anti-viral state but at the same time exacerbating the disease severity is still puzzling and warrant further dissection of IRF9 functions.

The novel SARS-CoV-2 also uses multiple mechanisms to hamper host IFN responses (91). During earlier episodes of coronavirus epidemic such as SARS-CoV and MERS-CoV, it was reported that expression of type I IFN (IFN-I) and other pro-inflammatory cytokines are usually suppressed for their successful pathogenesis (92, 93). A recent study have demonstrated that IRF9 among many other pro-inflammatory genes hold a high significance during immune related COVID-19 response (94). Another study have reported that IRF9 have a protective function in CNS and its deficiency could trigger severe neurological disease (95). The authors could show that IRF9 knockout mice brain shows calcification with massive inflammation and neurodegeneration (95). Interestingly, they have performed their experiments in cultured glial cells and showed that in IRF9-deficient glial cells, IFN-α can be more detrimental *via* inducing the expression of IFN-γ-like genes (95).

In our experiments with S-exo treated human microglia, we observed the similar situation where IRF9 levels have been downregulated. Our data helps in explaining the reason behind accelerated neurological aberration during SARS-CoV-2 even when there is lack of active viral load. Since we were inclined to understand the neurological perturbations/CNS damage as a bystander impact of SARS-CoV-2, we have carried out our experiments on human microglia; the executor of immune responses in CNS (96). We intended to mimic the neurological anomalies observed especially when peak viremia of SARS-CoV-2 have passed but host plasma is still enriched with dysregulated circulating host cellular factors.

Our study is presenting a novel bystander pathway for causing neuroinflammatory damage. This pathway begins with Spike induced exosome secretion, loaded with miR-148a and miR-590. Internalized miR-148a and miR-590 targets USP33 and IRF9 respectively. Here, miR-590 can directly target IRF9 while miR-148a suppresses the USP33 expression levels in human microglia. USP33 is a deubiquitinase by function, hence protects its target from being polyubiquitinated and degraded. We have identified for the first time, in our knowledge, that deubiquitination of IRF9 is regulated by USP33. Therefore, any perturbation in cellular USP33 levels is going to directly impact the turnover time of IRF9 into the cell. This regulatory cascade of exosomes carried miR-148a targeting USP33, influencing IRF9 stability and ultimately the inflammatory gene expression profile of microglia becomes an enigmatic double edged sword. As reflected by previous studies, IRF9 deficiency is especially disastrous in glial cells (95). It can shift the cytokine expression profile towards inflammatory and neurodegeneration like phenotype in CNS.

**Figure 7:**
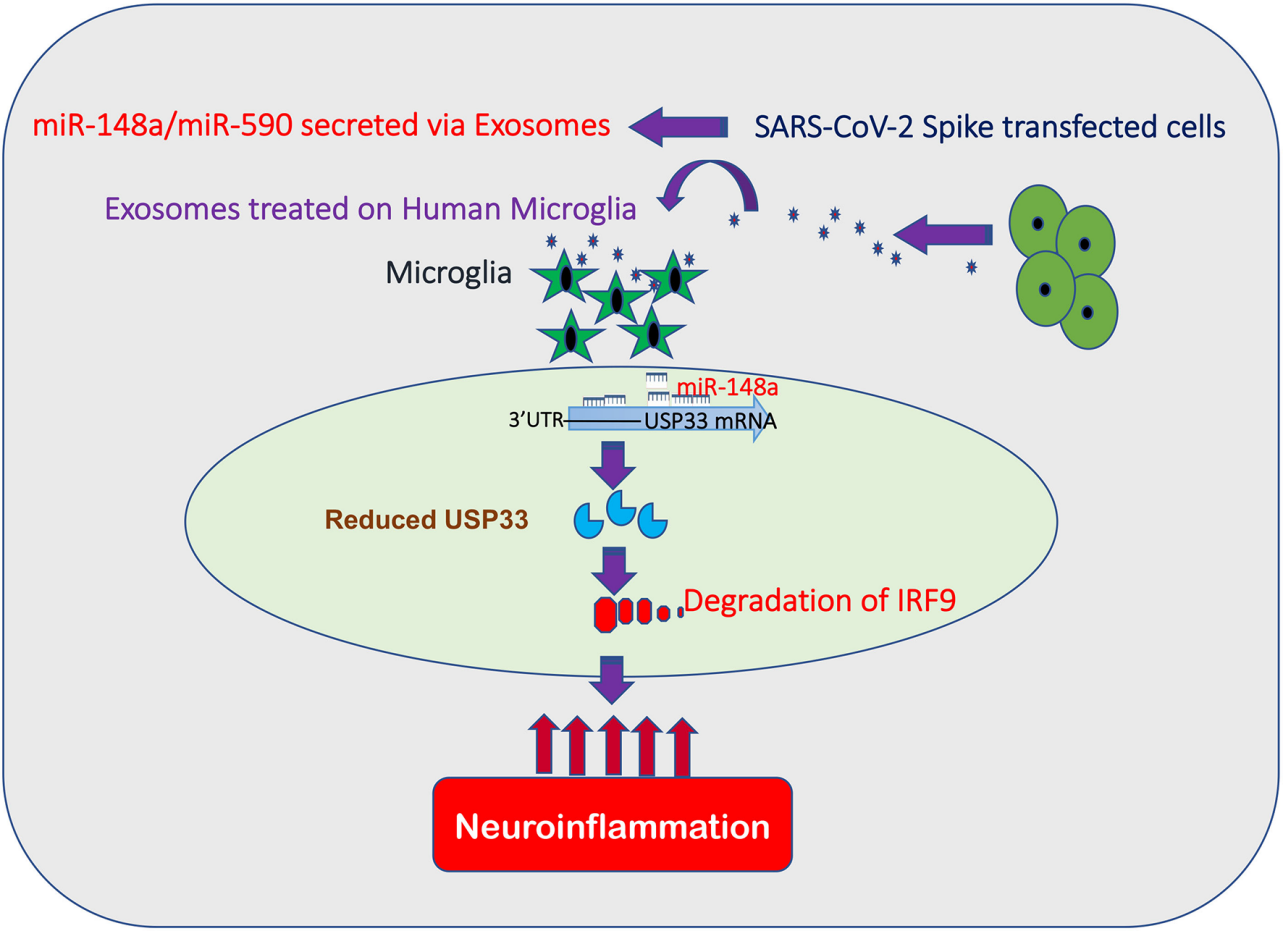
SARS-CoV-2 Spike targets USP33-IRF9 axis *via* Exosomal miR-148a to activate Human Microglia. Schematic flow diagram representing the mechanistic route utilized by SARS-CoV-2 Spike protein to target USP33-IRF9 axis *via* modulating exosomal miR-148a to activate the human microglial cells.

In conclusion, we are demonstrating in this study for the first time that stability and levels of IRF9, an enigmatic inflammation regulator, is controlled *via* a deubiquitinase USP33 in human microglia. Disruption of USP33-IRF9 axis stimulate the non-canonical activation of pro-inflammatory genes from microglia and lead to severe neuroinflammation inside CNS (**Figure 7**). Since a dysregulated host immune response and inflammation have been held responsible for cytokine storm/multiple organ dysfunctions and death during SARS-CoV-2 infection, a deeper understanding of immunoregulatory pathways are urgently needed. Predominant use of Spike gene as candidate epitope in vaccine development also warrants some detail investigation regarding its impact on host immune response and other safety concerns since few episodes of vaccine administration have reported some unexpected negative outcomes on host bodies. This study therefore have explored the impact of SARS-CoV-2 Spike gene and how it can modulate the host immune responses. Our study have thrown some light on new immune regulatory check points in human microglia which need to be explored further for finding new treatment modalities to combat SARS-CoV-2 neuropathogenesis.

## Data Availability Statement

The original contributions presented in the study are included in the article/supplementary material. Further inquiries can be directed to the corresponding authors.

## Author Contributions

RM and ACB conceived the research idea. RM has performed most of the experiments. Data acquisition, data analysis, and manuscript writing have been done by RM. Manuscript is read, edited, and supervised by AB. All authors contributed to the article and approved the submitted version.

## Funding

RM is a recipient of DST INSPIRE Faculty award and research grant as number DST/INSPIRE/04/2016/000169.

## Conflict of Interest

The authors declare that the research was conducted in the absence of any commercial or financial relationships that could be construed as a potential conflict of interest.

## Abbreviations

IRF9, Interferon regulatory factor 9; USP33, Ubiquitin Specific Peptidase 33; TNFα, Tumor necrosis Factor α; NF-κB, Nuclear factor- κB; IFN-β, Interferon β; CNS, Central Nervous System; COVID-19, Coronavirus disease 2019; ACE2, Angiotensin Converting Enzyme 2; HEK293T, Human embryonic kidney cells.; 3’UTR, 3’ Un-translated region; ATF3, Activating transcription factor 3; USP42, Ubiquitin Specific Peptidase 42; USP7, Ubiquitin Specific Peptidase 7; DUBs, deubiquitinase enzyme; JEV, Japanese Encephalitis Virus; ISRE, Interferon-stimulated response element; ISGF3, Interferon-stimulated gene factor 3; CHME3, human microglia cell line.
